# P01-022 – MEFV gene mutations registered to infevers

**DOI:** 10.1186/1546-0096-11-S1-A26

**Published:** 2013-11-08

**Authors:** A Berdeli, S Nalbantoglu, D Tigli, I Demirel, M Atan

**Affiliations:** 1 Molecular Medicine Laboratory, EGE UNIVERSITY, Izmir, Turkey

## Introduction

Familial Mediterranean Fever (FMF) is the most common hereditary autoinflammatory disorder characterized by fever and abdominal pain. 16p13.3 chromosomally located MEFV gene has been responsible for disease outcome and its protein product, Pyrin, is the key regulator protein of inflammasome complex which leads to IL-1B production and inflammation.

## Objectives

Here, we aimed to identify responsible MEFV gene mutations in clinically prediagnosed FMF patients and link to typical phenotype.

## Methods

Bidirectional DNA Sequencing analysis of MEFV gene in all coding exons and exon-intron boundaries was performed in Turkish patients clinically pre-diagnosed as FMF consulted in Ege University School of Medicine between years 2009-2013 (n=8000) and in healthy control group individuals (n=250). For patients who were mutation negative in screened exons, exons 1, 4, 6, 7, 8, and 9 were also analysed.

## Results

14 novel missense and nonsense mutations were investigated and registered to INFEVERS (http://fmf.igh.cnrs.fr/ISSAID/infevers) p.R151S (c.453G>C); p.S154P (c.460T>C); p.S166L(c.497C>T); p.S179N (c.536G>A); p.R241K (c.722G>A), p. P350R(c.1049C>G), p.E456D (c.1368A>C) ; p.Y471X (c.1413C>A); p.R501C (c.1501C>T); p.S503C (c.1508C>G); p.I506V (c.1516A>G), p.K695N (c.2085G>C); p.L709R (c.2126T>G) and p. I729V(c.2185A>G). In phenotypic correlation, p.Arg241Lys and p.Ser166Leu mutations were linked to recurrent fever; and p.Ile506Val and p.Leu709Arg missense mutations were seen as atypical FMF phenotype while the remaining ones were correlated well with FMF clinical implications.

## Conclusion

Identification of responsible mutations has great importance in disease maintanence, follow-up and proper treatment. It is recommended to prevent overlook uncommon pathogenic mutations in routine techniques via whole gene mutation analysis.

## Disclosure of interest

None declared.

